# Review of non-clinical risk models to aid prevention of breast cancer

**DOI:** 10.1007/s10552-018-1072-6

**Published:** 2018-09-03

**Authors:** Kawthar Al-Ajmi, Artitaya Lophatananon, Martin Yuille, William Ollier, Kenneth R. Muir

**Affiliations:** Division of Population Health, Health Services Research and Primary Care, Faculty of Biology, Medicine and Health, Centre for Epidemiology, The University of Manchester, Manchester, M139 PL UK

**Keywords:** Assessment risk tool, Calibration, Discrimination, Risk factors, Risk prediction, Concordance and E/O statistics

## Abstract

A disease risk model is a statistical method which assesses the probability that an individual will develop one or more diseases within a stated period of time. Such models take into account the presence or absence of specific epidemiological risk factors associated with the disease and thereby potentially identify individuals at higher risk. Such models are currently used clinically to identify people at higher risk, including identifying women who are at increased risk of developing breast cancer. Many genetic and non-genetic breast cancer risk models have been developed previously. We have evaluated existing non-genetic/non-clinical models for breast cancer that incorporate modifiable risk factors. This review focuses on risk models that can be used by women themselves in the community in the absence of clinical risk factors characterization. The inclusion of modifiable factors in these models means that they can be used to improve primary prevention and health education pertinent for breast cancer. Literature searches were conducted using PubMed, ScienceDirect and the Cochrane Database of Systematic Reviews. Fourteen studies were eligible for review with sample sizes ranging from 654 to 248,407 participants. All models reviewed had acceptable calibration measures, with expected/observed (E/O) ratios ranging from 0.79 to 1.17. However, discrimination measures were variable across studies with concordance statistics (C-statistics) ranging from 0.56 to 0.89. We conclude that breast cancer risk models that include modifiable risk factors have been well calibrated but have less ability to discriminate. The latter may be a consequence of the omission of some significant risk factors in the models or from applying models to studies with limited sample sizes. More importantly, external validation is missing for most of the models. Generalization across models is also problematic as some variables may not be considered applicable to some populations and each model performance is conditioned by particular population characteristics. In conclusion, it is clear that there is still a need to develop a more reliable model for estimating breast cancer risk which has a good calibration, ability to accurately discriminate high risk and with better generalizability across populations.

## Introduction

Breast cancer is the most common cancer among females in high-, middle- and low-income countries and it accounts for 23% of all new female cancers globally [1, 2]. While there has been a significant reduction in mortality, incidence rates have continued to rise [3]. Breast cancer incidence rates are high in North America, Australia, New Zealand, and Western and Northern Europe. It has intermediate levels of incidence in South America, Northern Africa, and the Caribbean but is lower in Asia and sub-Saharan Africa [1].

Early detection of breast cancer improves prognosis and increases survival. Mammographic imaging is the best method available for early detection [4] contributing substantially in reducing the deaths caused by breast cancer [5]. Unfortunately mammography mass screening still leads to some levels of over-diagnosis and over-treatment [6]. As yet routine mammography screening is not readily available globally, particularly in some developing countries [7, 8]. This is supported by the observations that for every million adult women there are only four mammogram screening machines in Sudan has four mammogram machines, whereas Mexico has 37 and Canada has 72 [9]. Under these circumstances, it is clearly more appropriate to prioritize access to mammographic screening or other targeted interventions (such as tamoxifen chemoprevention) for higher-risk individuals who could be identified using a sensitive and specific risk prediction model [10]. Such risk prediction models are individualized statistical methods to estimate the probability of developing certain medical diseases. This is based on specific risk factors in currently healthy individuals within a defined period of time [11]. Such prediction models have a number of potential uses such as planning intervention trials, designing population prevention policies, improving clinical decision-making, assisting in creating benefit/risk indices and estimating the burden cost of disease in population [10].

A general case can also be made for using risk models for certain diseases. For example, their use can allow the application of risk-reducing interventions that may actually prevent the disease in question. If their application can be based on use of existing health records this will avoid increasing levels of anxiety in at least low to moderate risk individuals. The National Cancer Institute of the USA (NCI) has confirmed that the application of “risk prediction” approaches has an extraordinary chance of enhancing “The Nation’s Investment in Cancer Research” [12]. This provides an explanation for the rapid increase in the number of models now being reported in the literature [11, 13]. It is clear that not all developed models are valid or can be widely used across populations. The minimum performance measures required for a useful and robust risk prediction model in clinical decision making are discrimination and calibration [14].

We recognize that risk models are increasingly now being used as part of a “triage” assessment for mammography and/or for receipt of other more personalized medical care. There is a growing interest in applying risk prediction models as educational tools.

The models developed can differ significantly with regard to; the specific risk factors that are included; the statistical methodology used to estimate, validate and calibrate risk; in the study design used; and in the populations investigated to assess the models. These differences make it essential that any assessment of model usefulness takes into account both their internal and external validity. Here, we focus on the reliability, discriminatory accuracy and generalizability of breast cancer risk models that exclude clinical (any variable which needs physician input e.g., presence of atypical hyperplasia) and any genetic risk factors. Accurate assessment of risk using easily acquired data is essential as a first stage of tackling the rising burden of breast disease globally. Well-validated models with high predictive power are preferable although this is not the case for all models. The usability of any model is dependent on the purpose the model will be used for and its target populations [15]. Furthermore, it has been suggested that adapting existing predictive models to the local circumstances of a new population rather than developing a new model for each time is a better approach [16].

This review focuses on breast cancer risk predicting models that incorporated modifiable risk factors and/or factors that can be self-reported. Such models could be applied as an educational tool and potentially used to advice at risk individuals on appropriate behavioural changes.

## Methods

### Databases

The following databases were searched for all related publications (up to July 2016): PubMed (https://www.ncbi.nlm.nih.gov/pubmed/); ScienceDirect (http://www.sciencedirect.com/); the Cochrane Database of Systematic Reviews (CDSR) (http://www.cochranelibrary.com/). Terms used for the search were “assessment tool, assessment model, risk prediction model, predictive model, prediction score, risk index, breast cancer, breast neoplasm, breast index, Harvard model, Rosner and Colditz model, and Gail model”. Risk models were retrieved based on any study design, study population or types of risk factors.

**Fig. 1:**
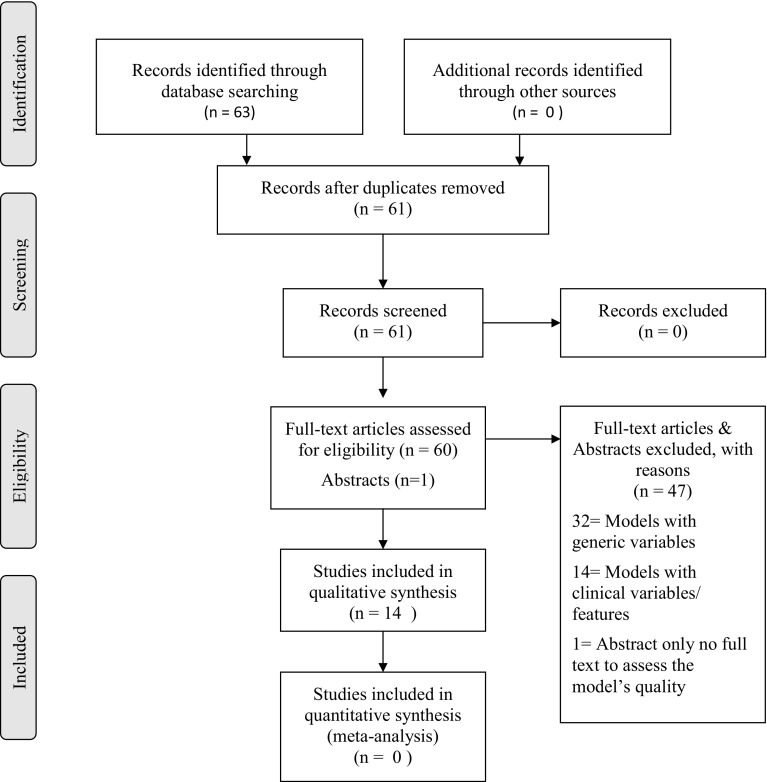
Identification of eligible risk models using PRISMA flowchart

A Preferred Reporting Items for Systematic Reviews and Meta-Analyses (PRISMA) approach was applied for selecting reviewed articles [17]. A total of 61 genetic and non-genetic breast cancer risk models were identified and then filtered to include only risk models with non-clinical factors (Fig. 1). These models contain variables which are considered to be modifiable and/or self-reported by the respondents. For this review, 14 studies were eventually considered to be eligible. No literature reviews were found on breast cancer risk models solely focusing on epidemiological risk factors although all the selected reviews summarized generic composite risk models. The literature search was extended to include publications relating to systematic reviews and meta-analyses; this did not reveal any appropriate publications.

### Confidence in risk factors

Details relating to the degree of confidence in variables used as risk factors in the risk models were taken from the Harvard report [18]. The degree of confidence was categorized as either:

- definite (an established association between outcome and exposure where chance, bias [systematic error], confounders [misrepresentation of an association by unmeasured factor/s] are eliminated with significant confidence)
- probable (an association exists between the outcome and the exposure where chance, bias, confounders cannot be eliminated with sufficient confidence—inconsistent results found with different studies)
- possible (inconclusive or insufficient evidence of an association between the outcome and the exposure)

## Results

### Potential risk factors included in breast cancer non-clinical predictive models

The variables used in the 14 models under review and specifies the degree of confidence (definite, probable or possible) in those variables as risk factors for breast cancer based on the current literature are summarized in Table 1.

**Table 1:** Breast cancer risk factors included in the 14 models

Name of model	Gail [37]		Rosner [42]	Rosner [25]	Colditz [50]	Ueda [38]	Boyle [39]	Lee [36]	Novotny [24]	Gail [32]	Matsuno [51]	Banegas [40]	Pffeifer [31]	Park [23]	Lee [33]	Effect	Level of evidence
Basic characteristics																	
Age	Yes		Yes	Yes	Yes		Yes	Yes	Yes	Yes		Yes			Yes	Increased risk	Definite
Ethnicity											Yes					Jewish increased risk	Definite
Height					Yes											Increased risk	Definite
Weight					Yes											Increased risk in post-menopausal	Probable
BMI					Yes	Yes	Yes						Yes	Yes	Yes		Probable
Alcohol intake					Yes		Yes	Yes					Yes	Yes		Increased risk	Probable
Smoking								Yes						Yes		Increased risk	Possible
Physical activity							Yes							Yes	Yes	Decreased risk	Possible
Diet							Yes									Decreased risk	Probable
Hormonal and reproductive factors																	
Age at menarche	Yes		Yes	Yes	Yes	Yes	Yes		Yes	Yes	Yes	Yes		Yes	Yes	Increased risk	Definite
Age at first live birth	Yes		Yes	Yes	Yes	Yes	Yes	Yes	Yes	Yes	Yes	Yes	Yes	Yes	Yes	Increases risk	Definite
Age at subsequent birth			Yes	Yes												Increases risk	Definite
Age at menopause			Yes	Yes	Yes								Yes	Yes	Yes	Increased risk	Definite
Hormone replacement therapy use					Yes		Yes						Yes	Yes		Increases risk	Definite
Oral contraceptive use					Yes				Yes					Yes		Increases risk	Definite
Breast feeding								Yes						Yes		Decreases risk	Probable
Pregnancy														Yes		Decreases risk	Possible
Parity					Yes								Yes			Decreases risk	Definite
Children number					Yes										Yes	Decreases risk	Possible
Menopause type					Yes											Surgical menopause reduces risk	Possible
Menstrual regularity								Yes								Menstrual regularity and duration—inconsistent results	Possible
Menstrual duration								Yes							Yes		Possible
Menopausal status													Yes		Yes	Post-menopause increases risk	Possible
Gestation period															Yes	Increases risk	Possible
Family history of breast and/or ovarian cancer or diseases																	
Family history of breast cancer		Yes			Yes	Yes	Yes	Yes	Yes		Yes		Yes	Yes	Yes	Increases risk	Definite
First-degree relatives with breast cancer		Yes			Yes					Yes		Yes				Increases risk	Definite
Age of onset of breast cancer in a relative							Yes									Increases risk	Probable
Benign breast disease					Yes				Yes				Yes			Increases risk	Probable
History of breast biopsies		Yes		Yes					Yes	Yes	Yes	Yes		Yes		Increases risk	Definite
Mammogram															Yes	Increases risk	Probable
Summary of risk factors included in each model																	
Definite factors	5		5	6	10	3	5	3	6	3	5	5	5	7	5	Max of 10 and min of 3 factors	
Probable factors	0		0	0	4	1	3	2	1	0	1	0	3	3	2	Max of 4 and min of 0 factors	
Possible factors	0		0	0	2	0	2	3	0	0	0	0	1	3	5	Max of 5 and min of 0 factors	
Total factors	5		5	6	16	4	8	8	7	3	5	5	9	13	12	Max of 16 and min of 3 factors	

Age, age at first birth, age at menarche, family history of breast cancer, and self-reported history of biopsies were the most common variables used amongst the 14 models selected. These variables are considered as definite risk factors for developing breast cancer [18]. Other additional variables were observed in fewer models. These included ethnicity (Jewish—definite), definite hormonal replacement therapy, diet (some probable and others possible), physical activity (possible), height (definite), weight (probable- for pre-menopausal women and definite for post-menopausal women). Among pre-menopausal females, weight is considered to be a protective factor [19]. In contrast amongst post-menopausal women, weight is considered to be a risk factor [20–22] as is parity, oral contraceptive pill use (definite), pregnancy history, timing and type of menopause (definite), menstrual regularity (possible), menstrual duration and gestation period (probable), smoking (possible), mammogram screening (probable) and age of onset of breast cancer in a relative (definite).

The largest number of definite factors included in a model (*n* = 10 variables) was seen in the study reported by Colditz and Rosner [18]. This was followed by studies by reported by Park [23], Novotny [24] and Rosner [25]. We evaluated the number of the definite, probable and possible variables in the models to compare their performance based on the type and number of the variable included.

### Evaluation measures of the risk models

The most important measures used to assess the performance of the models were considered to be as follows:

- Calibration (reliability): the E/O statistic measures the calibration performance of the predictive model. Calibration involves comparing the expected versus observed numbers of the event using goodness-of-fit or chi square statistics. A well-calibrated model will have a number close to 1 indicating little difference between the E and O events. If the E/O statistic is below 1.0 then the event incidence is underestimated, while if the E/O ratio is above 1.0 then incidence is overestimated [14, 26].
- Discrimination (precision): the C statistic (Concordance statistic) measures the discrimination performance of the predictive model and corresponds to the area under a receiver operating characteristic curve. This statistic measures how efficiently the model is able to discriminate affected individuals from un-affected individuals. A C-statistic of 0.5 indicates no discrimination between individuals who go on to develop the condition and those who do not. In contrast, a C-statistic of 1 implies perfect discrimination [27, 28]. Good discrimination is important for screening individuals and for effective clinical decision making [10].
- Accuracy: is tested by measuring of ‘sensitivity’, ‘specificity’, ‘positive predictive value’ (PPV) and negative predictive value (NPV). All of these terms are defined in Table 2. These measures indicate how well the model is able to categorize specific individuals into their real group (i.e., 100% certain to be affected or unaffected). Accuracy is equally important for both individual categorisation and for clinical decision making. Nevertheless, even with good specificity or sensitivity, low positive predictive values may be found in rare diseases [10] as the predictive values also depend on disease prevalence. With high prevalence, PPV will increase while NPV will decrease [29].
- Utility: this evaluates the ease with which the target groups (public, clinicians, patients, policy makers) can submit the data required by the model. Utility evaluation assesses lay understanding of risk, risk perception, results interpretation, level of satisfaction and worry [30]. This evaluation usually uses surveys or interviews [26].

**Table 2:** Formulas used to calculate the accuracy of the model

Term	Definition	Equation
Sensitivity	Probability of a test will indicate ‘positive’ among those with the disease	(TP)/(TP + FN)
Specificity	Probability of a test will indicate ‘negative’ among those without the disease	(TN)/(TN + FP)
Positive predictive value	Probability of a patient having disease when test is positive	(TP)/(TP + FP)
Negative predictive value	Probability of a patient not having disease when test is negative	(TN)/(FN + TN)

*TP* True positive, *TN* true negative, *FP* false positive, *FN* false negative

**Fig. 2:**
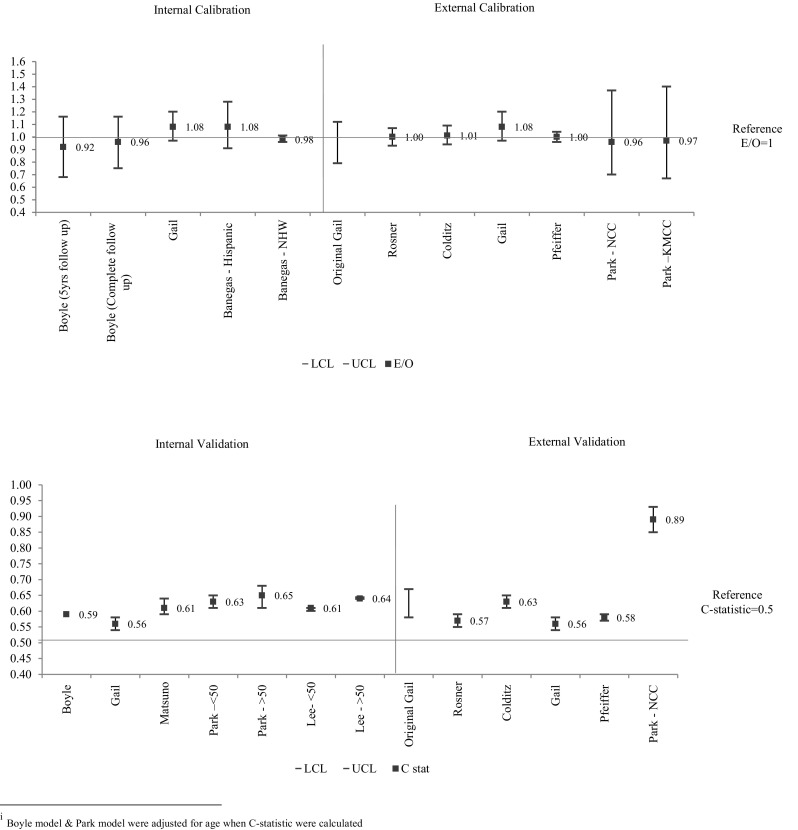
Calibration and discrimination performances of the 13 breast cancer risk models

Calibration and discrimination were the most common measures used to assess the breast cancer risk models under review and these measures are summarized in Fig. 2. Internal calibration was performed in just three of the 14 models with values ranging from 0.92 to 1.08. These calibration values represented a good estimate of the affected cases using these models. For external calibration, six of the 14 models used an independent cohort. Rosner [25] and Pfeiffer [31] reported the highest with E/O values of 1.00 and followed by Colditz [18] with an E/O of 1.01.

The C-Statistic values measuring internal discrimination ranged across studies from 0.61 to 0.65. The Park [23] model achieved the best outcome (C-Statistic = 0.64). Additionally, Park [23] showed the highest value with a C-Statistic of 0.89 when applied to subjects recruited from the NCC (National Cancer Centre) screening program. The lowest C-Statistic (0.56) was observed in the Gail model [32]. Overall, this demonstrates that the models have better calibration than discrimination. Accuracy was only evaluated in the Lee model [33]. Sensitivity, specificity and overall accuracy were calculated. The values indicate low accuracy with values ranging from 0.55 to 0.66 (Table 3).

In qualitative research relating to the impact and utility [34] of the Harvard Cancer Risk Index (HCRI) [18], nine focus groups (six female, three male) showed good overall satisfaction with HCRI. Participants appreciated both the detailed explanation and the updated inclusion of risk factors. On the other hand, some participants criticized the absence of what they considered to be important factors (e.g., environmental factors and poverty). Some participants believed that some of the factors on which subjects had been assessed might cause anxiety. It is also noted, however, that the case has been made that such anxiety provides motivation for action to mitigate risk [35].

### Overview of current models

All the models described (except for Lee et al. 2004) [36] are extended versions of either the Gail model or the Rosner and Colditz model (Tables 4, 5). The Gail model developed in 1989 [37] was the first risk model for breast cancer and included the following variables: age, menarche age, age at first birth, breast cancer history in first-degree relatives, history of breast biopsies and history of atypical hyperplasia. The range of calibration of the Gail modified models was E/O = (0.93–1.17) and the discrimination range was C-Statistics = (0.56–0.65). This indicates that these models are well calibrated, although discrimination could be improved.

**Table 3:** Summary of the evaluation measures of the risk models

Model	Calibration			Discrimination			Accuracy	Utility
	Derived model	Internal	External	Derived model	Internal	External	Sensitivity, specificity, PPV, NPV	
Gail [37]			0.79–1.12			0.58–0.67		
Rosner [42]	–	–	–	–	–		–	–
Rosner [25]		–	1.00 (0.93–1.07)^d^	–		0.57 (0.55–0.59)^d^		
Colditz [50]	–	–	1.01 (0.94–1.09)^d^	–		0.64 (0.62–0.66)^d^	–	Good^e^
Ueda [38]	–	–	–	–	–		–	–
Boyle [39]^a^		(a) 0.96 (0.75–1.16) cohort1 (b) 0.92 (0.68–1.16) cohort2	–	0.59			–	–
Lee [36]	–	–	–	–	–		–	–
Novotny [24]	–	–	–	–	–		–	–
Gail [32]	–	1.08 (0.97–1.20)	0.93 (0.97–1.20)^f^	–		0.56 (0.54–0.58)^f^	–	–
Matsuno [51]	1.17 (0.99–1.38)				0.614 (0.59–0.64)		–	–
Banegas [40]^b^	–	(a) 1.08 (0.91–1.28); Hispanic (b) 0.98 (0.96–1.01); NHW	–	–	–	–	–	–
Pfeiffer [31]			1.00 (0.96–1.04)			0.58 (0.57–0.59)		
Park [23]^c^	–	–	(a) 0.97(0.67–1.40); KMCC (b) 0.96 (0.70–1.37); NCC	–	(a) 0.63 (0.61–0.65) < 50 years (KMCC) (b) 0.65 (, 0.61–0.68) ≥ 50 years (KMCC)	(a) 0.61(0.49–0.72); KMCC (b) 0.89(0.85–0.93); NCC	–	–
Lee [33]					Overall: 0.62 (0.620–0.623) Under 50: 0.61 (0.60–0.61) Above 50: 0.64 (0.63–0.64)		(a) Sensitivity Overall: 0.55 (0.54–0.56) < 50: 0.61 (0.60–0.62) > 50:0.59 (0.59–0.60) (b) Specificity Overall: 0.66 (0.65–0.67) > 50: 0.58 (0.57–0.59) < 50:0.64 (0.63–0.65) (c) Accuracy Overall: 0.60 (0.60–0.61) > 50:0.59 (0.59–0.60) < 50:0.61 (0.61–0.62)	–

^a^Boyle [39] used two cohorts for calibration (1-cohort with complete follow-up and 2-cohort with 5 years of follow-up at most)

^b^Banegas [40] used two cohorts for calibration (1-Hispanic and 2-non-Hispanic white (NHW))

^c^Park [23] used two cohorts for calibration and discrimination, using two Korean cohorts: 1-the Korean Multi-center Cancer Cohort (KMCC) and 2-National Cancer Centre (NCC) cohort

^d^[49]

^e^[52]

^f^[11]

**Table 4:** Characteristic summary of the reviewed breast cancer risk models

Author/model	Study design	Participants	Ethnicity	Outcome	Statistical method	Effect estimates	Sample size	Risk factors considered in the models	Age target	Stratification
Gail [37]	Case–control	White American females from the Breast Cancer Detection Demonstration Project (BCDDP)	American–Caucasian	Invasive breast cancer + in situ carcinoma	unconditional logistic regression	Relative risk	2,852 cases 3,146 controls	Age at menarche, age at first live birth, number of previous biopsies, and number of first-degree relatives with breast cancer	Any age	None
Rosner [42]	Cohort	Registered nurses	American–Caucasian	Invasive breast cancer	Poisson regression	Cumulative incidence	2,341 cases, 91,523 controls	Age, age at all births, menopause age, menarche age	30–55 years	Number of births
Rosner [25]	Cohort	Registered nurses	American–Caucasian	Invasive breast cancer	Poisson regression	Relative risk	2,249 cases, 89,132 controls	Menarche age, first live birth age, subsequent births age, menopause age	Any age	None
Colditz [50]	Cohort	General women	American–Caucasian	Invasive breast cancer	Poisson regression	Cumulative incidence	1,761cases 56,759 controls	Benign breast disease, use of HRT, weight, height, menopausal type, and alcohol intake	Women aged 30–55 years	None
Ueda [38]	Case–control	General women	Japanese–Asian	Invasive breast cancer	Conditional logistic regression	Relative risk	376 cases 430 controls	Menarche, first birth age, family history, and BMI in post-menopausal women	Any age	Menopausal status
Boyle [39]	Case–control	General women	Italian–Caucasian	Invasive breast cancer	Conditional logistic regression	Absolute + relative risk	2,569 cases 2,588 controls	Menarche age, first birth age, alcohol intake, family history, age of diagnosis in relatives, and one of the two diet scores. BMI and HRT were included only for women older > 50	23–74 years (cases) 20–74 years (controls)	Age (< 50 and > 50)
Lee [36]	Case–control	1-General women 2-Well educated (nurse/teacher)	Korean–Asian	Invasive breast cancer	Hosmer–Lemeshow goodness of fit	Probability	384 cases 270 controls	With hospitalized controls: family history, menstrual regularity, total menstrual duration, first full-term pregnancy age, breastfeeding duration while with nurse/teacher controls: age, menstrual regularity, drinking status, smoking status	Age at least 20 years	None
Novotny [24]	Case–control	General women	Czeck females–Caucasian	Invasive breast cancer	Unconditional Logistic regression	Relative risk	4,598 matched pairs	Age at birth of first child, family history of breast cancer, No. of previous breast biopsy, menarche age, parity, history of benign breast disease	Age matched	None
Gail [32]	Case–control	General women	African American	Invasive breast cancer	Conditional logistic regression	Absolute + relative risk	1,607cases 1,647 controls	Menarche age, No. of affected mother or sisters, No. of benign biopsy	35–64 years	Age (< 50 and > 50)
Matsuno [51]	Case–control	General women	Asian and Pacific Islander American	Invasive breast cancer	Conditional logistic regression	Absolute + relative + attributable risks	589 cases 952 controls	Menarche age, age at first live birth, No. of biopsies, family history, ethnicity	Any age	Ethnicity
Banegas [40]	Longitudinal study	General women	Hispanic	Invasive breast cancer	Cox proportional hazards regression	Relative risk	6,353 cases 128,976 controls	Age, age at first live birth, menarche age, No. of first-degree relatives with breast cancer, No. of breast biopsies	Post-menopausal participants aged ≥ 50	None
Pfeiffer [31]	Prospective study	White over 50 years old	White and non-Hispanic Caucasian	Invasive breast cancer	Cox proportional hazards regression	Relative and attributable risks	7,695 cases 240,712 controls	BMI, oestrogen and progestin MHT use, other MHT use, parity, age at first birth, pre-menopausal, age at menopause, benign breast diseases, family history of breast or ovarian cancer, and alcohol consumption	50 and above	None
Park [23]	Case–control	General women	Korean–Asian	Invasive breast cancer	Unconditional Logistic regression	Absolute risk	3,789 cases 3,789 controls	Family history, menarche age, menopausal status, menopause age, pregnancy, first full-term pregnancy age, No. of pregnancies, breastfeeding duration, OC usage, HRT, exercise, BMI, smoking, drinking, No. of breast examinations	Any age	Age (< 50 and > 50)
Lee [33]	Case–control	General women	Asian	Invasive breast cancer	Conditional logistic regression		2,291 cases and 2,283 controls	First full-term pregnancy age, children No., menarche age, BMI, family history, menopausal status, regular mammography, exercises, oestrogen exposure duration, gestation period, menopause age	Any age	Age (< 50 and > 50)

**Table 5:** Models reviewed in this article

	Title	Size of study	Population	First author	References
Included in this review	Projecting individualized probabilities of developing breast cancer for white females who are being examined annually	2,852 cases 3,146 controls	Caucasian	Gail 1989	[37]
	Reproductive risk factors in a prospective study of breast cancer: the Nurses’ Health Study	2,341 cases, 91,523 controls	Caucasian	Rosner 1994	[42]
	Nurses’ health study: log-incidence mathematical model of breast cancer incidence	2,249 cases, 89,132 controls	Caucasian	Rosner 1996	[25]
	Cumulative risk of breast cancer to age 70 years according to risk factor status: data from the Nurses’ Health Study	1,761cases 56,759 controls	Caucasian	Colditz	[50]
	Estimation of individualized probabilities of developing breast cancer for Japanese women	376 cases 430 controls	Asian	Ueda	[38]
	Contribution of three components to individual cancer risk predicting breast cancer risk in Italy	2,569 cases 2,588 controls	Caucasian	Boyle	[39]
	Determining the Main Risk Factors and High-risk Groups of Breast Cancer Using a Predictive Model for Breast Cancer Risk Assessment in South Korea	384 cases 270 controls	Asian	Lee	[36]
	Breast cancer risk assessment in the Czech female population–an adjustment of the original Gail model	4,598 matched pairs	Caucasian	Novotny	[24]
	Projecting individualized absolute invasive breast cancer risk in African American women	1,607cases 1,647 controls	African	Gail	[32]
	Projecting individualized absolute invasive breast cancer risk in Asian and Pacific Islander American women	589 cases 952 controls	Asian	Matsuno	[51]
	Evaluating breast cancer risk projections for Hispanic women	6,353 cases 128,976 controls	Hispanic	Banegas	[40]
	Risk Prediction for Breast, Endometrial, and Ovarian Cancer in White Women Aged 50 y or Older: Derivation and Validation from Population-Based Cohort Studies	42,821 cases 114,931 controls	White, non-Hispanic women aged 50+	Pfeiffer	[53]
	Korean risk assessment model for breast cancer risk prediction	3,789 cases 3,789 controls	Asian	Park	[23]
	Computational Discrimination of Breast Cancer for Korean Women Based on Epidemiologic Data Only	2,291 cases and 2,283 controls	Asian	Lee	[33]
Excluded from this review	[54–101]				

Ueda et al. [38] modified the Gail model by including age at menarche, age at first delivery, family history of breast cancer and BMI in post-menopausal women, as risk factors in his model for Japanese women. However, as with the original Gail model, no validation was performed. In the Boyle model [39], more factors were included such as alcohol intake, onset age of diagnosis in relatives, one of the two diet scores and BMI and HRT. This results in calibration with E/O close to unity and less acceptable discrimination of C-stat = 0.59. The Novotny model [24] added the number of previous breast biopsies performed on a woman and her history of benign breast disease. However, no validation assessment was performed for this model. Newer models [32, 40, 41] included the number of benign biopsies. This resulted in acceptable calibration but less acceptable discrimination (Gail [32]: E/O = 0.93; C-stat = 0.56; Matsuno: E/O = 1.17, C-statistic = 0.614; and Banegas E/O = 1.08). Park et al. [23] included menopausal status, number of pregnancies, duration of breastfeeding, oral contraceptive usage, exercise, smoking, drinking, and number of breast examinations as risk factors. This model has an E/O = 0.965; C-stat = 0.64. However, the C-statistic reported from the external validation cohort was high compared to the original C-statistic. They reported a C-statistic of 0.89 using the NCC cohort. This discrepancy was claimed to be caused by the population characteristics (participants were 30 years and above, recruited from cancer screening program, from a teaching hospital in an urban area) [23]. In the same year, Pfeiffer et al. [23] developed a model where parity was considered as a factor and had E/O of 1.00 and a C-statistic of 0.58. The later Gail model published in 2007 used logistic regression to derive relative risks. These estimates are then combined with attributable risks and cancer registry incidence data to obtain estimates of the baseline hazards [32].

The Rosner and Colditz model of 1994 [42] was based on a cohort study of more than 91,000 women. The model used Poisson regression (rather than logistic regression as in the Gail model). The variables were as follows: age, age at all births, menopause age, and menarche age. This model was not validated. A new version in 1996 [25] included one modification (current age was excluded) and gave an E/O = 1.00 and a C-statistic = 0.57. In 2000, Colditz et al. [18] modified the model with risk factors for: benign breast disease, use of post-menopausal hormones, type of menopause, weight, height, and alcohol intake. This model gave an E/O = 1.01; C-statistic = 0.64.

Lee et al. [36] used two control groups: a “hospitalised” group and a nurses and teachers group. The risk factors in the hospitalized controls were as follows: family history, menstrual regularity, total menstrual duration, age at first full-term pregnancy, and duration of breastfeeding. The risk factors in the nurses/teachers control group were as follows: age, menstrual regularity, alcohol drinking status and smoking status. This model was not based on Gail or Rosner and Colditz. Hosmer–Lemeshow goodness of fit was used to assess model fit which had a *p* value = 0.301 in (hospital controls) and *p* value = 0.871 in (nurse/teacher controls). No calibration or discrimination measures were reported.

Lee [33] used three evaluation techniques to assess the discrimination and the accuracy of their model: support vector machine, artificial neural network and Bayesian network. Of the three, support vector machine showed the best values among the Korean cohort. However, accuracy and discrimination were less acceptable in this model.

In summary, calibration performance is similar between models (Modified Gail and modified Rosner, Colditz), yet modified Gail models showed better discrimination performance with the C-statistic of the Park model being 0.89.

## Discussion

There is increasing interest among clinicians, researchers and the public in the use of risk models. This makes it important that we fully evaluate model development and application. Each risk model should be assessed before it can be recommended for any clinical application. Performance assessment should involve the use of an independent population [43] separate from the population used to build the model. We have reviewed breast cancer risk models that include non-genetic and non-clinical risk factors but exclude clinical risk factors. By using PubMed, ScienceDirect, Cochrane library and other research engines, 14 models met these criteria. The most recent model examined was developed in 2015 [33]. Most models were based on two earlier risk models developed over 20 years ago—the Gail model [37] and the Rosner and Colditz model [42]. The modified versions of these two original models varied in the risk factors included and the estimation methods used. In 2012, there were two literature reviews published which analysed breast cancer risk prediction models [11, 28]; however, our review focuses particular on modifiable risk factors and/or self-reported factors and we have updated the models published after 2012 [23, 31, 33].

Most models with modifiable risk factors included report acceptable calibration, with E/O close to 1 but less acceptable discrimination with C-statistic close to 0.5. Calibration and validation were improved when more definite factors were included. A possible explanation for less acceptable discrimination performance could be the inclusion of weaker evidence-based factors (probable and possible risk factors). All the models had combinations of probable and possible factors with no single model restricted to the inclusion of the definite factors.

Various factors affect model performance. Inclusion of less significant factors is likely to occur in studies with small sample sizes [11, 28]. Some important clinical risk factors were not included and this may affect the model’s final performance [44]. Breast cancer heterogeneity may also contribute to poor performance as different cancer types may have different risk factors [11]. Most of the models included in this review did not stratify breast cancer into its subtypes during model development. Rosner and Colditz however evaluated the model’s performance based on breast cancer subtypes (ER±, PR ± or HR2±) and concluded that risk factors vary according to the subtypes [45, 46]. Finally, even when strong risk factors are included in a model, significant increases in C-statistic have not been seen [47].

Model performance statistics were affected by the criteria used to stratify the analysis. Four models were stratified by age (below 50 and above 50). One model was further stratified by menopausal status [38], one by ethnicity [41] and one by number of births [42]. Breast cancer risk models could be improved if appropriate factors were used to stratify the population. For example, pre-menopausal and post-menopausal females have different risk factors in breast cancer development. The models that applied menopausal status have some limitation in that this may not be applicable to women who have had hysterectomy. For example, in the US, hysterectomy is the second most common procedure performed and the likelihood of oophorectomy varies by age at hysterectomy [48]. Hence, completion of risk assessment outside of a clinical setting is problematic as women may be challenged to define their menopausal status. Even though the overall performance of these models appears to be moderate in differentiating between cases and non-cases, they may still serve as a good educational tool as part of cancer prevention. Utility evaluation assesses the public’s knowledge of breast cancer risk factors rather well and could be used to promote cancer risk reduction actions.

A significant limitation in the development of risk models is the absence of consensus standards for defining and classifying a model’s performance. For example what is the level of good or acceptable calibration or measures of discrimination? what are acceptable measures of specificity and sensitivity in diagnostic/prognostic/preventive models? how close to unity should calibration and discrimination be for a model to be considered valid? what is the utility cut-off in each type of model? All of these questions are hard to answer without global agreement. However, this lack of consensus is understandable as these values vary depending on the type of the model type (diagnostic, prognostic, preventive), goal (clinical tool, educational tool, screening tool), targeted audience (public, high-risk patients, patients visiting the clinic) and the disease itself and its types or subtypes (such as breast cancer, familial breast cancer, lobular/ductal/invasive/in situ carcinoma breast cancer). This suggests that the closer value of E/O and C-statistics to 1, the better model performance. Such a pragmatic attitude permits us to begin to focus on improving the availability of effective risk reduction actions.

Furthermore, some of the models reviewed cannot be applied to some of the populations as the risk factors may vary between different populations. For example, alcohol consumption would not be applicable to Muslim women. We recommend that researchers develop a more reliable and valid breast cancer risk model which has good calibration, accuracy, discrimination and utility where both internal and external validations indicate that it can be reliable for general use. In order to improve our models, the following should be considered: (1) the model type (diagnostic, prognostic, preventive), goal (clinical tool, educational tool, screening tool), targeted audience (public, high-risk patient), (2) inclusion of definite risk factors while incorporating the clinical and/or genetic risk factors where possible, (3) dividing the model into disease subtypes, age and menopausal status, (4) ensuring that a model is developed that can be validated externally.

## Abbreviations

CDSR: Cochrane Database of Systematic Reviews
ROC: Area under a receiver operating characteristic curve
AUC: Area under a receiver operating characteristic curve
NPV: Negative predictive value
PPV: Positive predictive value
TP: True positive
TN: True negative
FP: False positive
FN: False negative
NCC: National Cancer Centre cohort
KMCC: Korean Multi-center Cancer Cohort
NHW: Non-Hispanic white
NCI: National Cancer Institute
E/O: Expected/observed
